# Why Emergency Physicians Should Advocate for Suspension of Title 42 Restrictions on Asylum for US Immigrants

**DOI:** 10.5811/westjem.2022.11.59464

**Published:** 2023-02-22

**Authors:** Kevin Durgun, Emmeline Ha, Natalie Kirilichin, Janice Blanchard

**Affiliations:** The George Washington University, Department of Emergency Medicine, Washington, DC

In an interview with *Time* magazine in 2019, Violeta Monterroso described her fears and inability to return to Guatemala after a gang attempted to extort her and threatened the lives of her family. “They kill the people and kill their children. The first thing is to have security for them,” Monterroso said of her kids, “that nothing bad happens to them.”^1^

Undocumented immigrants frequently present to emergency departments (ED) in the United States. Conversely, they may not present until late in their disease course for fear of discovery and deportation. Either way, the status of undocumented immigrants is squarely “in the lane” of emergency medicine and deserves advocacy on par with other issues that plague our underserved patient population. Title 42 expulsion is a provision of the Public Health Service Act of 1944. In short, it is a policy that enables the federal government to restrict an individual’s entry to the US due to public health concerns.^2^ It was first implemented in March 2020 by the Trump administration as part of the broad range of actions to address the coronavirus disease 2019 (COVID-19) pandemic.

Such experiences like those Ms. Monterroso described are not isolated events, with one study quantifying 23% of asylum seekers fleeing gang violence and 34% fleeing violent family members.^3^ The countries from which many of these individuals emigrate often have civilian violent death rates comparable to countries at war.^4^ As physicians, we have an obligation to recognize the health and human realities that our patients face, as well as the consequences that immigration policies have on these already marginalized people.

As emergency physicians, we must also consider what effects immigration has on our EDs. Current narratives perpetuate the myth of migrants as disproportionately using emergency services. Objective measures do not support this. Recent studies demonstrate that refugees used emergency services less frequently than non-refugee controls.^5^ Undocumented immigrants are noted to use emergency services less frequently than US citizens and other migrant groups.^6^ Interventions connecting undocumented immigrants to primary care services have been demonstrated to reduce ED visits.^7^ Migrants face unique challenges when presenting for emergency care. For example, they may be hesitant to report labor abuses, sexual violence, or physical assault, or be unable to pay for outpatient primary care or specialty services.^8^ Despite these challenges, one study estimates that healthcare expenditures for immigrants, undocumented or authorized, cost less per capita compared to US-born patients.^9^ We should not accept policies that curtail legal immigration and instead push toward unauthorized migration and more complicated emergency care.

The primary argument for implementing Title 42 was to protect the US public from COVID-19 exposures resulting from potential cases crossing the border. One of the populations most affected by Title 42 were asylum-seekers who came to the US southern border via Mexico to seek entry on grounds of safety or fear of persecution in their native country. Prior to implementation of Title 42, the policy of Migrant Protection Protocol (MPP), commonly referred to as “Remain in Mexico,” instituted new procedures to hold them in Mexico during their legal asylum proceedings. With the start of the COVID-19 pandemic, Title 42 was used to effectively end nearly all migration and asylum claims processing at the US-Mexico border. The US Border Patrol estimates there was a dramatic increase to 1,040,220 Title 42 expulsions in 2021, compared to 197,043 expulsions in 2020.^10^ Most migrants were unable to file an asylum claim. For those who have filed claims through MPP, a backlog in processing could lead to wait times of almost four years.^11^

Title 42 was continued under the Biden Administration. An attempt to lift it was blocked in May 2022 by a federal judge in Louisiana after a lawsuit was filed by the attorneys general of Arizona and 24 other states. His decision cited a failure to follow procedure to obtain public comment and evaluate strains to local healthcare, education, and law enforcement.^12^ A decision is pending from the US Court of Appeals for the Fifth Circuit. In November 2022, a federal judge on the US District Court for the District of Columbia ordered the lifting of Title 42, citing that it violated the administrative procedures act and that the US Centers for Disease Control and Prevention had failed to consider alternatives.^13^ A stay was issued shortly thereafter, giving the Biden Administration until December 21 to prepare for the transition. A definitive end for Title 42 remains elusive.

Title 42 is problematic for several reasons. Despite its implementation as a public health order, multiple leading health and human rights organizations have said there is no public health or rigorous data that supports the claim that these restrictions have mitigated the COVID-19 pandemic in the US.^14^ In fact, former White House medical advisor Dr. Anthony Fauci asserted that immigration is not a principal driver of COVID-19 transmission and that expelling immigrants is not the solution to outbreaks.^15^

Since the onset of the pandemic, the US never completely closed its borders to foreign travel. International travel has been allowed since November 2021. Special immigration exceptions have been made for Ukrainian and Afghani citizens, while barring Haitian, Venezuelan, and other Central and South American asylum seekers. COVID-19 has been widespread in the US since 2020. Title 42 is increasingly being supported from an immigration policy perspective, rather than from a public health perspective. We should be wary of the dangers of such political actions, especially since clear racial and ethnic biases have developed.

Furthermore, Title 42 and other strict border policies put asylum seekers at health risk in two key ways. First, broad restrictions on legal immigration hold vulnerable people in crowded, under-resourced, and dangerous situations indefinitely. In complying with Title 42, asylum seekers are kept in Mexico or other countries without fulfillment of previously mandated medical screening.^16^ There is little protection from violence,^17^ and many develop severe mental health disorders from the both the emotional and physical trauma suffered.^18^ Migrants cite barriers to healthcare in Mexico including costs,^19^ food insecurity,^20^ and crowded shelters with infectious disease outbreaks.^21^

Secondly, when faced with these dangers of waiting indefinitely under Title 42, asylum seekers may instead choose to cross increasingly dangerous walls and subsequently encounter militarized border guards or perilous natural obstacles including the Sonoran Desert and the Rio Grande River. An example is the San Diego-Tijuana border wall, which after being raised from 17 feet to 30 feet was associated with a five-fold increase in the number of hospital admission for border wall falls at the trauma center of the University of California, San Diego (67 fall admissions prior to compared to 375 after the height change).^22^ Other threats include the risk of death from environmental exposures such as dehydration or drowning, with 609 migrant bodies found as of July 19, 2022, compared to 566 in all of 2021.^23^ The worst human smuggling-related incident in modern US history occurred when 51immigrants were found dead in a truck trailer in San Antonio, TX, on June 27, 2022.^2^

Emergency physicians are uniquely sensitive to the effects of public health policy. By allowing the weaponization of public health toward immigration policy, we are conceding a major component of our profession to the realm of politics. Regardless of political affiliations, the concession of public health policies runs contrary to our role to advocate for our patients, within *and* outside the ED. Emergency physicians should advocate for the suspension of Title 42 as a harmful and unnecessary policy that directly and indirectly affects emergency patients, healthcare, and society.
